# A pilot clinical evaluation of oral mucosal dryness in dehydrated patients using a moisture‐checking device

**DOI:** 10.1002/cre2.145

**Published:** 2019-02-07

**Authors:** Yosuke Fukushima, Yoshie Sano, Yuta Isozaki, Mao Endo, Taketo Tomoda, Tomohisa Kitamura, Tsuyoshi Sato, Yoshito Kamijo, Yoshiyuki Haga, Tetsuya Yoda

**Affiliations:** ^1^ Department of Oral and Maxillofacial Surgery Faculty of Medicine, Saitama Medical University Japan; ^2^ Emergency Medical Center and Poison Center Saitama Medical University Hospital Japan

**Keywords:** dehydration, oral moisture‐checking device, oral mucosal dryness

## Abstract

This study included 30 patients (17 males and 13 females; mean age, 73.7 ± 13.1 years) who were diagnosed with dehydration based on vital signs, skin symptoms, and blood test findings by emergency medicine physicians. First, the attending physician of our department measured oral mucosal dryness. Subsequently, the emergency medicine physician blindly divided the severity of dehydration into three stages according to clinical findings and blood test results. In this study, the oral moisture‐checking device (Mucus®; Life Co., Ltd., Saitama, Japan) was used to measure the oral mucosal dryness. We examined the oral moisture level for each dehydration severity level and the correlations of each severity level of dehydration with the measured values. Spearman's correlation coefficient (Medcalc version 11.3 for Windows) was used for statistical analysis. *P* < 0.05 indicated significant differences. Twenty‐six patients were diagnosed with dry mouth, and a moderate negative correlation was found between the severity of dehydration and oral moisture degree (*r* = −0.686). The correlation coefficient for the relationship between oral moisture degree and severity of dehydration was −0.686, indicating a negative correlation (*P* < .05). These results suggest that the oral mucosal dryness may be a useful index of dehydration severity.

## 1 INTRODUCTION

Dehydration can lead to death in severe cases; thus, early diagnosis is important. However, in dehydrated patients, clinical symptoms and clinical laboratory findings are often inconsistent, especially in the elderly. Dehydration can progress rapidly, and subjective findings can be poor due to dementia or cerebral vascular disease; additionally, it can be difficult to collect blood data in institutionalized patients (Yoshida et al., 2007).

To solve these problems, a simple and noninvasive method to measure dehydration should be developed. Recent studies have reported the utility of salivary viscosity (Yoshida et al., 2007), saliva osmotic pressure (Fortes et al., 2015), capillary refill time (Shimizu et al., 2012), and axillary dryness (Kinoshita et al., 2013; Shimizu et al., 2012) as diagnostic indicators. However, there is limited evidence that these devices evaluate these parameters conveniently and numerically, and there are also concerns regarding their objectivity and reproducibility. Dry mouth is often associated with dehydration. McGee et al. (McGee et al., 1999) reported that 85% of dehydrated patients experienced dry mouth, which corroborated Shimizu et al.'s four findings that dry mouth is one of the most common clinical findings in dehydrated patients. Given the availability of oral moisture‐checking devices (Fukushima et al., 2013; Fukushima et al., 2017; Murakami et al., 2009; Osailan et al., 2012; Takahashi et al., 2006; Yamada et al., 2005), we hypothesized that dry mouth measures by use of such device may provide a rapid and precise indication of dehydration. In this pilot clinical study conducted in a medical emergency setting, we established the relationship between body dehydration determined clinically and the dryness of the oral mucosa measured by use of oral moisture‐checking devices.

## 2 MATERIALS AND METHODS

The Institutional Review Board of Saitama Medical University provides ethical approval to conduct this pilot clinical study (approval number: 15–087).

A convenience sample of *n* = 30 adults were recruited among patients referred for acute treatment at the Department of Emergency Medicine and Poison Center, Saitama Medical University Hospital. Patients with an existing diagnosis of dry mouth and those diagnosed with Sjögren's syndrome or oral mucosal abnormalities were not invited to partake in this study. All study participants received verbal explanations regarding measurements of oral wetness and signed an informed consent. After completion of the initial treatment for dehydration, the study participants were again explained about the protection of privacy and personal information and provided the freedom to continue or withdraw their consent.

Dehydration was diagnosed by emergency medicine physicians using vital signs, skin symptoms, and blood tests (creatinine, blood urea nitrogen [BUN], uric acid, serum sodium, and serum chloride serum potassium). The patients were categorized into one of three categories of dehydration (mild:1, moderate:2, and severe:3) according to clinical findings and blood test results, independent of the degree of oral moisture.

**Figure 1:**
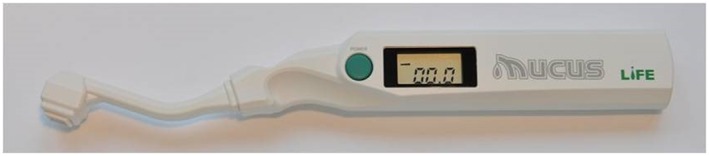
Oral moisture‐checking device (Mucus®, Life Co., Ltd.)

The extent of oral moisture was measured using an oral moisture‐checking device (Mucus®;serial number 401398 Life Co., Ltd., Saitama, Japan; Figure 1). This device measures the electrostatic capacity on the basis of impedance generated by connecting high‐frequency waves supplied by a 5‐V battery to plus and minus comb‐shaped electrodes depicted on the surface of a 7.2‐mm^2^ sensor in 2 s. In addition to the water content of the oral mucosal surface, the electrostatic capacity reflects intramucosal water content to a depth of about 50 μm. Mucus® has received a manufacturing and marketing approval as a body composition analyzer (approval number: 22200BZX00640000), by the Pharmaceutical and Medical Devices Agency of Japan. The sensitivity and specificity values are close to 80%. Oral moisture values range from 0 to 99.9, and values of ≥29.6, 28.0–29.5, and ≤27.9 are defined as normal, borderline dry mouth, and dry mouth, respectively (Fukushima et al., 2017).

**Figure 2:**
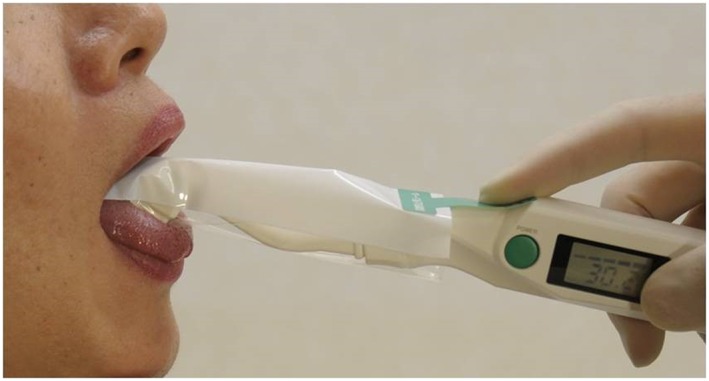
Measurement of oral moisture degree of the lingual mucosa with sensor cover

Oral moisture was measured on the center of the lingual mucosa approximately 10 mm from the tip of the tongue (Figure 2). A disposable polyethylene cover was applied to the sensor, which was manually applied to the measurement site at a pressure of approximately 200 g, as practiced beforehand with a manometer. The measurements were done in triplicate, and the median values were used (Fukushima et al., 2007).

### 2.1 Statistical analysis

The correlations of the extent of dehydration with the oral moisture degree and blood test results were examined. Spearman's correlation coefficient (Medcalc version 11.3 for Windows) was used for statistical analysis, and *P* < 0.05 indicated statistical significance.

## 3 RESULTS

**Figure 3:**
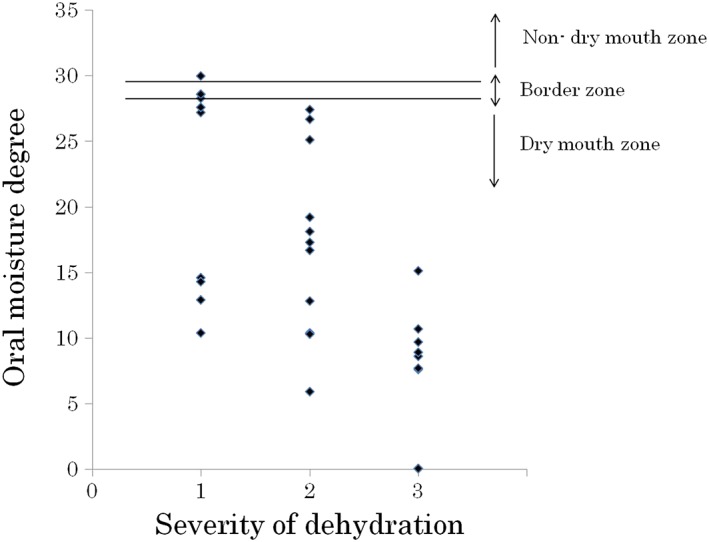
Oral moisture degree in each severity of dehydration

**Figure 4:**
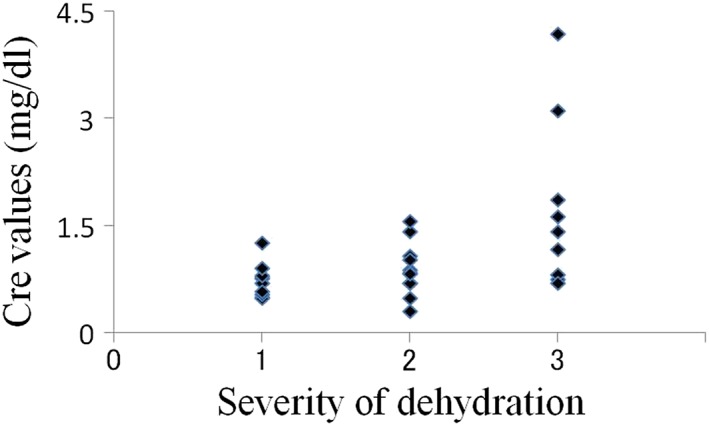
Creatinine values in each severity of dehydration

**Figure 5:**
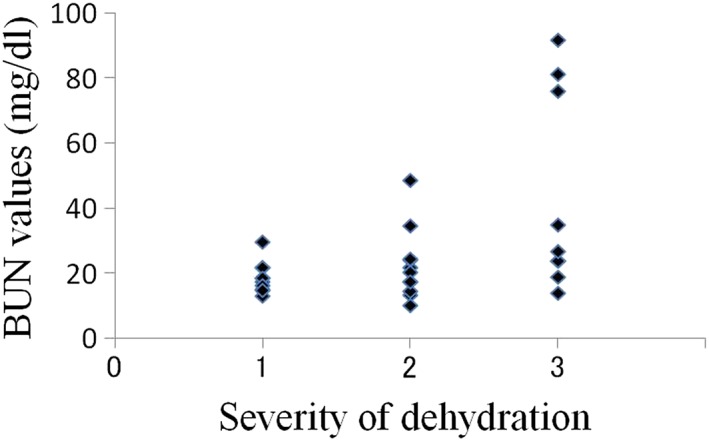
Blood urea nitrogen values in each severity of dehydration

**Figure 6:**
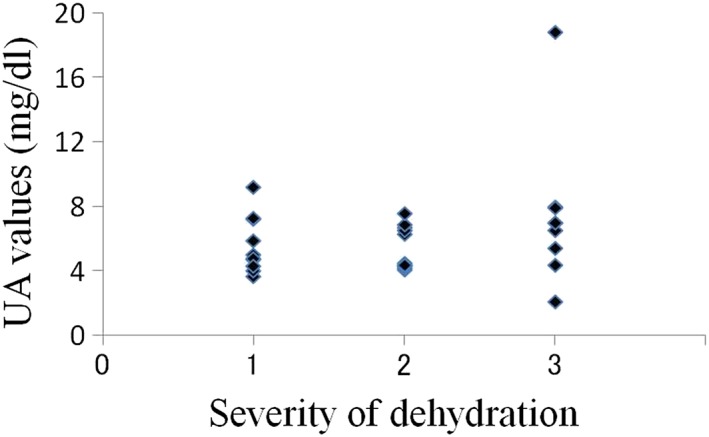
UA values in each severity of dehydration

**Figure 7:**
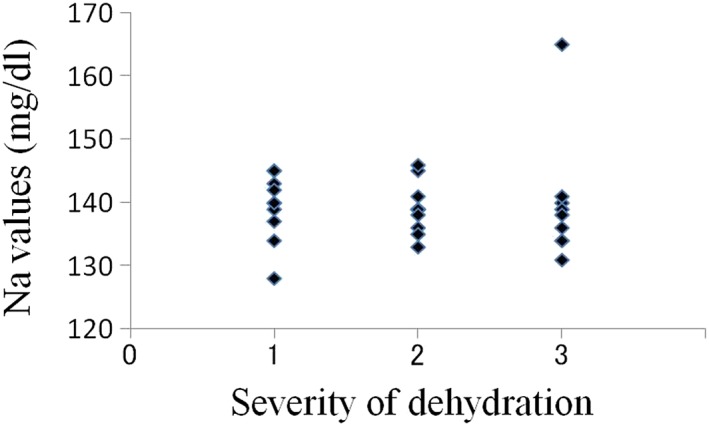
Na values in each severity of dehydration

**Figure 8:**
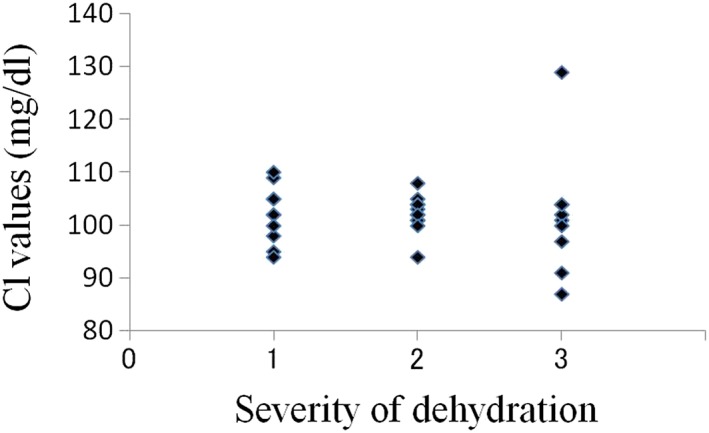
Cl values in each severity of dehydration

**Figure 9:**
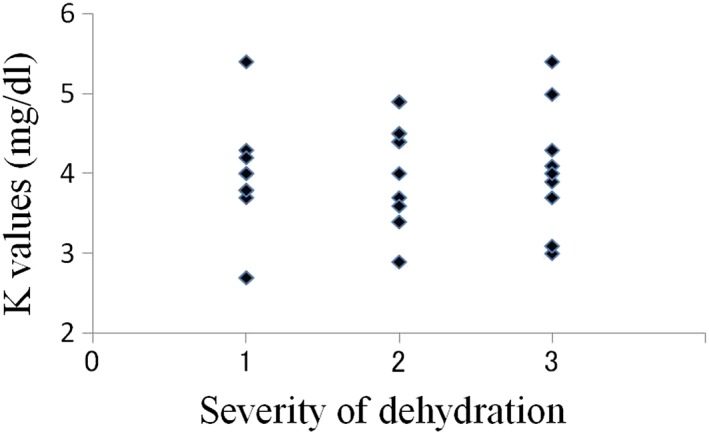
K values in each severity of dehydration

The average oral moisture value in the 30 patients was 16.0 ± 8.78 (Table 1). The average values in patients with mild (10 patients), moderate (11 patients), and severe dehydration (nine patients) were 22.3, 17.1, and 7.6, respectively. In total, 26 patients were diagnosed with dry mouth using our criteria, and all nine patients with severe dehydration had oral moisture values of less than 15 (Figure 3). In the relations between creatinine value, BUN value, and severity of dehydration, there was a tendency that the number of plots with high value increased with progression of the degree of dehydration; however, such tendency was not seen in UA, Na, Cl, and K values (Figures 4, 5, 6, 7, 8, 9).

**Table 1:** Patient characteristics

Sex	Male	17 cases
	Female	13 cases
Mean age		73.7 ± 12.9
Mean value of blood tests	Cr	1.1 ± 0.79 mg/dl
	BUN	25.5 ± 20.14 mg/dl
	UA	6.1 ± 2.84 mg/dl
	Na	139.3 ± 6.33 mEq/l
	Cl	102.2 ± 7.07 mEq/l
	K	4.0 ± 0.70 mEq/l
Severity of dehydration	Mild	10 cases
	Moderate	11 cases
	Severe	9 cases
Mean of oral moisture degree		16.0 ± 8.78(0.03–30.0)

*Note*. BUN: blood urea nitrogen.

The mean creatinine, BUN, uric acid, serum sodium, serum chloride, and serum potassium levels and BUN/creatinine value ratio in the patients were 1.1 ± 0.79 mg/dl, 25.5 ± 20.14 mg/dl, 6.1 ± 2.84 mg/dl, 139.3 ± 6.33 mEq/l, 102.2 ± 7.07 mEq/l, 4.0 ± 0.70 mEq/l, and 27.8, respectively. Regarding the BUN/creatinine ratio, the values were <20, 20–24.9, and ≥25 in 10, 4, and 16 patients, respectively.

Meanwhile, Spearman's correlation coefficient illustrated that the oral moisture value had a moderate negative correlation with the severity of dehydration (*P* < 0.05). Additionally, the correlation coefficients for the relationships of the severity of dehydration with the creatinine and BUN levels were 0.5 and 0.44, respectively, indicating moderately positive correlations (*P* < 0.05, Table 2).

**Table 2:** The correlation coefficients of severity of dehydration with each values

	Oral moisture degree	Cr	BUN	UA	Na	CL	K
Correlation coefficients	−0.686	0.5	0.44	0.21	−0.19	−0.14	0.11
*P* value	0.00003	0.0049	0.0014	ns	ns	ns	ns

*Note*. BUN: blood urea nitrogen.

## 4 DISCUSSION

The findings of the study indicated that dry mouth is a common symptom in patients with dehydration. Meanwhile, even patients with high‐oral moisture values exhibited dry mouth. One explanation for these findings was that some patients drank water before transportation to the hospital, potentially increasing oral mucosal dryness.

Regarding oral mucosal dryness, Okuyama et al. (Okuyama & Nishida, 2016) observed no difference between a control group and a group of patients with suspected dehydration. However, because the control group of their study consisted of elderly people with dementia who required nursing care, it was concluded that both groups likely exhibited dry mouth. In the current study, all patients with severe dehydration exhibited remarkable dry mouth, suggesting that oral mucosal moisture can be an index of dehydration.

A common index used to indicate dehydration is the BUN/creatinine ratio. A normal value is approximately 10.0, and higher values indicate dehydration. However, some papers defined values of more than 20.0 as indicating dehydration (Hodgkinson et al., 2003; Hoffman, 1991; Weinberg et al., 1994), whereas others used a cutoff of 25.0 (Stookey et al., 2005). In the current study, the mean BUN/creatinine ratio was 27.8. However, no significant correlation was observed between the severity of dehydration and the BUN/creatinine ratio, whereas the severity of dehydration was moderately correlated with the oral moisture degree. This revealed the difficulty of evaluating severity in dehydration. In this study, a significant moderate negative correlation with the severity of dehydration and oral moisture degree was shown. Taking this into consideration, it was suggested that measurement of oral moisture degree using with oral moisture device may be useful to evaluate the severity of dehydration conveniently.

The amount of water in the body decreases with dehydration. As a result, body fluid osmotic pressure increases, thirst center in the hypothalamus is stimulated, and thirsty feeling and dry‐mouth feeling occur (Hodgkinson et al., 2003; World Health Organization (WHO), 2003). Regarding the treatment of dehydration, it is critical to evaluate its progression and supply water to patients. It is known that dehydration results in the sensation of thirst (Suzuki & Kashimura, 2015). However, this sensation is subjective, especially in elderly people, and malfunction of the central control of thirst can cause this sensation to not appear despite dehydration (Hooper et al., 2014; Tsutsumi et al., 2003). Therefore, tools to measure oral moisture may be useful for screening for dehydration in elderly people and asymptomatic people, especially in nursing care facilities lacking medical personnel. Additionally, such devices would be useful for detecting dehydration and preventing heat strokes during athletic events.

The results of this study indicated that oral moisture degree of less than 20.0 can indicate dehydration, and emergency transport may be considered for people with values of less than 15.0. We next plan to clarify the diagnostic criteria for dehydration using oral mucosal dryness in studies with larger patient groups.

## CONFLICT OF INTEREST

None declared.

## STATEMENT FROM AUTHORS

This manuscript has not been published elsewhere, and it is not under consideration by another journal. The manuscript has been carefully reviewed by an experienced editor whose first language is English and who specializes in editing papers written by scientists whose native language is not English. All authors meet the authorship criteria detailed in the Authorship section of this guideline and that all authors are in agreement with the content of the manuscript.
